# Bursopentin (BP5) induces G1 phase cell cycle arrest and endoplasmic reticulum stress/mitochondria-mediated caspase-dependent apoptosis in human colon cancer HCT116 cells

**DOI:** 10.1186/s12935-019-0849-3

**Published:** 2019-05-16

**Authors:** Jing Li, Tian-xiang Li, Yao Ma, Yong Zhang, De-yuan Li, Hai-rong Xu

**Affiliations:** 1 Institute of Translational Medicine, Medical College, Yangzhou University, Yangzhou, 225009 People’s Republic of China; 2 Jiangsu Key Laboratory of Integrated Traditional Chinese and Western Medicine for Prevention and Treatment of Senile Diseases, Yangzhou University, Yangzhou, 225009 People’s Republic of China; 3 Department of Clinical Medicine, Kangda College of Nanjing Medical University, Lianyungang, 222000 People’s Republic of China; 4 Key Lab of Animal Disease Diagnosis and Immunology, Ministry of Agriculture, College of Veterinary Medicine, Nanjing Agricultural University, Nanjing, 210095 People’s Republic of China; 5 Jiangsu Co-Innovation Center for the Prevention and Control of Important Animal Infectious Diseases and Zoonoses, College of Veterinary Medicine, Yangzhou University, Yangzhou, 225009 People’s Republic of China

**Keywords:** Apoptosis, Endoplasmic reticulum stress, G1 cell cycle arrest, Mitochondrial pathway, Reactive oxygen species

## Abstract

**Background:**

Bursopentin (BP5) is a multifunctional pentapeptide found in the chicken bursa of Fabricius. Recent study indicated that BP5 significantly stimulates expression of p53 protein in colon cancer HCT116 cells. However, the effects and underlying mechanisms of BP5 on HCT116 cell proliferation remain largely unclear.

**Methods:**

Analyses of cell viability, cell cycle arrest as well as apoptosis were performed to study the actions of BP5 on HCT116 cells. Western blot analyse was assayed to measure the cell cycle-related and apoptosis-related proteins. Specific siRNAs targeting IRE1, ATF-6, and PERK were used for IRE1, ATF-6, and PERK knockdown, respectively. Cellular reactive oxygen species (ROS) were detected using a H_2_DCF-DA green fluorescence probe. Cytosolic free Ca^2+^ concentrations and mitochondrial membrane potential (ΔΨm) were measured using Fluo-3 AM and JC-1 stains, respectively.

**Results:**

BP5 possessed strong inhibitory effects on the cell growth and induced apoptosis in HCT116 cells. Mechanistically, BP5 arrested the cell cycle at G1 phase by increasing p53 and p21 expression and decreasing cyclin E1-CDK2 complex expression. BP5 treatment dramatically activated the endoplasmic reticulum (ER) stress-mediated apoptotic pathway, as revealed by the significantly enhanced expression of unfolded protein response (UPR) sensors (IRE1α, ATF6, PERK) as well as downstream signaling molecules (XBP-1s, eIF2α, ATF4 and CHOP), and by the significantly altered the BP5-induced phenotypic changes in IRE1, ATF6, and PERK knockdown cells. Additionally, BP5-induced ER stress was accompanied by the accumulation of cytosolic free Ca^2+^ and intracellular ROS. Furthermore, BP5 treatment resulted in the increase of Bax expression, the decrease of Bcl-2 expression and the reduction of ΔΨm, subsequently causing a release of cytochrome c from the mitochondria into the cytoplasm and finally enhancing the activities of caspase-9 and -3. In addition, z-VAD-fmk, a pan-caspase inhibitor, markedly rescued BP5-induced cell viability reduction and reduced BP5-induced apoptosis.

**Conclusions:**

Our present results suggest that BP5 has an anticancer capacity to arrest cell cycle at G1 phase and to trigger ER stress/mitochondria-mediated caspase-dependent apoptosis in HCT116 cells. Therefore, our findings provide insight into further investigations of the anticancer activities of BP5.

**Electronic supplementary material:**

The online version of this article (10.1186/s12935-019-0849-3) contains supplementary material, which is available to authorized users.

## Background

Human colon cancer is listed as one of the deadliest diseases and is the third leading cause of death from cancer [1]. Current chemotherapeutic drugs are effective in the treatment of diseases, but they are related to severe clinical toxicities and the development of drug resistance of cancer cells. For this reason, it is a huge challenge for developing new cytotoxic drugs [2–4]. The use of naturally occurring substances have been considered to be an effective and less toxic approach in the treatment of various diseases, including many human cancers [5–7]. Among many chemical protective substances, the naturally occurring agents are well known for their structural diversity and have been playing an encouraging role in drug discovery [8]. Therefore, several chemotherapeutic drugs developed from natural sources have been studied and are currently being used or are being investigated for cancer treatment.

Bursopentin (BP5, Cys-Lys-Arg-Val-Tyr) is a naturally occurring pentapeptide that is endogenously synthesized and found in the chicken bursa of the Fabricius (BF) [9]. Several studies have suggested that BP5 is multifunctional. For instance, a previous study showed that BP5 can exhibits immunomodulator effects on T and B lymphocytes [9]. It possesses the functions to stimulate humoral and cellular immune responses in chickens and mice [9, 10]. It was also found that BP5 has the ability to attenuate the immune function of dendritic cells, which are considered as a major target for immunomodulators [11]. Moreover, BP5 has been demonstrated to possess antioxidant activity and protect living organisms from oxidative stress via decreasing intracellular ROS generation [12, 13]. It has been suggested that BP5 may be used as a new antioxidant therapy to combat the oxidative stress [14]. More recently, it was reported that BP5 significantly enhances p53 luciferase activity and stimulates expression of p53 protein in HCT116 cells. By constructing a T7 phage display cDNA library, and using gene microarray, the differentially expressed genes associated with various pathways were identified, of which 25 pathways were involved in immune responses and oncogenic processes, including the p53 signalling pathway in DT40 cells [15]. However, no information is available on the inhibitory ability of BP5 on the growth of HCT116 colon cancer cells.

Thus, the purpose of this study was to research whether BP5 has any ability to inhibit cancer cell growth and elucidate any mechanisms that might underlie this process in colon cancer HCT116 cells.

## Materials and methods

### BP5 preparation

BP5 was achieved from a commercial company (Bootech, Shanghai, China). The peptide purity of BP5 was greater than 98%. Using an E-Toxate Limulus LPS assay kit (Sigma), synthetic BP5 was assayed to rule out the possibility of lipopolysaccharide (LPS) contamination. Only LPS-uncontaminated peptides were used.

### Cell culture

HCT116 cells and two immortalized normal cell lines (human keratinocyte HaCaT and mouse fibroblast NIH/3T3) were gained from the China Cell Bank of Type Culture Collection of (Shanghai, China). All these cells were incubated in DMEM with 10% foetal calf serum, 100 U/mL streptomycin (KeyGEN Biotech, Nanjing, China) and 100 U/mL penicillin (KeyGEN Biotech, Nanjing, China).

### Cell viability assay

Cell viability was analysed using the CCK-8 Kit (Beyotime, Shanghai, China). First, HCT-116, HaCaT and NIH/3T3 cells were cultured in 96-well plates at a seeding density of 1 × 10^4^ cells/mL. All cells were incubated with the indicated doses of BP5 for 24, 48 or 72 h after allowing 24 h for attachment. Then, the CCK-8 solution was added (10 μL/well) and the samples were cultured for another 2 h. The OD value of each well was analysed by an auto microplate reader (BioTek Instruments, USA). Observation of cell morphologic alterations were carried out with a Nikon Eclipse TS200 microscope (Nikon Corp., Tokyo, Japan).

### Cell cycle assay

HCT116 cells were cultured with the indicated doses of BP5. After 24 h of incubation, all samples were collected, and the cells were gently fixed overnight by using 70% ice-cold ethanol. Next, all samples were further incubated with PI (40 μg/mL) (KeyGEN Biotech, Nanjing, China), Triton X-100 (0.1%) and RNase (0.1 mg/mL). After incubation at 37 °C for 30 min, all cultures were analysed using a flow cytometer (BD Biosciences, San Jose, CA, USA).

### Cell apoptosis assay

HCT116 cells were cultured with the indicated amount of BP5. After 24 h of incubation, all samples were collected, and the cells were washed twice using cold PBS. Cells were then resuspended, treated with FITC annexin V, cultured at 4 °C for 15 min, and further incubated with PI. The double stained samples were then analysed using a FACS flow cytometer (BD Biosciences, San Jose, CA, USA).

### Intracellular ROS assays

HCT116 cells (1 × 10^4^/mL) were cultured overnight in glass plates. The cells were then treated with or without BP5 (2, 4, 8 mM) for 24 h. Samples were stained using 2,7-dichlorodihydrofluorescein diacetate (H_2_DCF-DA) fluorescent probe (Sigma-Aldrich, St. Louis, USA). At 37 °C in the dark, the stained cells were further cultured for another 30 min. In the presence of ROS, the nonfluorescent H2DCF-DA was turned into the fluorescent 2′,7′-dichlorofluorescein (DCF). The observation of cell images was carried out by a fluorescence microscope. ImageJ software was used to calculate the mean fluorescence intensity [16].

### Measurement of cytosolic Ca^2+^

Fluo-3 AM (KeyGEN Biotech, Nanjing, China), the fluorescent Ca^*2*+^ indicator, was used to evaluate level changes of the cytosolic Ca^*2*+^. After cells were treated as described earlier, they were collected and stained with Fluo-3 AM. Following an additional 30 min incubation, all stained samples were immediately detected using flow cytometry.

### Evaluation of mitochondrial membrane potential (ΔΨm)

Briefly, 24 h after BP5 treatment, all samples were harvested, and stained with JC-1 (KeyGEN Biotech, Nanjing, China). At 37 °C in the dark, the stained cells were further cultured for another 20 min. Then, the stained cells were centrifuged, removed the supernatant, resuspended in a 1× incubation buffer and analysed on a FACS flow cytometer (BD Biosciences, San Jose, CA, USA). Thereafter, all result analyses were carried out by using FlowJo v7.6 software (Tree Star Inc, Ashland, OR).

### Small interfering RNA (siRNA) transfection

Specific siRNAs targeting IRE1, ATF-6, PERK and were used for IRE1, ATF-6, and PERK knockdown. IRE1, ATF-6 and PERK siRNAs were provided by GenePharma (Shanghai, China). Their targeting sequences were as follows: 5′-GCAAGAACAAGCUCAACUATT-3′ for IRE1; 5′-GUGAGCUACAAGUGUAUUATT-3′ for ATF-6; 5′-GUGGAAAGGUGAGGUAUAUTT-3′ for PERK. A non-targeting siRNA (si-NC, also synthesized by GenePharma) was employed as a negative control. Briefly, HCT116 cells were cultured and transiently transfected with 5 nM siRNAs using Lipofectamine 2000 (Invitrogen, Carlsbad, CA, USA). After an additional 4 h incubation, all samples were cultured with or without BP5 (8 mM) for 24 h. Then, all samples were prepared for the evaluation of cell viability, apoptosis and the protein expression levels of IRE1, ATF-6, and PERK.

### Western blot analysis

HCT116 cells were homogenized in the lysate buffer, and the total proteins were prepared. Using a commercial mitochondria/cytosol fractionation kit (Beyotime, Shanghai, China), the cytosolic and mitochondrial proteins were obtained separately. The cell cycle arrest-related proteins of p53, p21, Cyclin E1, and CDK2; the ER stress-associated proteins of p-IRE1α, IRE1α, p-PERK, PERK, ATF6, XBP-1s, p-eIF2α, eIF2, ATF4, and CHOP; and the apoptosis-associated proteins of Bax, Bcl-2, cytoplasmic cytochrome c (Cyto Cyt C), mitochondrial cytochrome c (Mito Cyt C), caspase-9 and -3 as well as cleaved caspase-3, were all evaluated by western blot analysis.

### Statistical analysis

Statistical analyses were performed using SPSS 17.0 (SPSS, Inc., Chicago, IL, USA) or GraphPad Prism 5.0 (GraphPad Software, San Diego, CA, USA) software. Data from the individual experiments were expressed as the mean ± SD. Statistical significance between the groups was determined with one-way analysis of variance (ANOVA) or Student’s t-test. *P *< 0.05 was considered to be a statistically significant difference.

## Results

### BP5 inhibits HCT116 cell growth but has no significant effects on the proliferation of HaCaT and NIH/3T3 cells

**Fig. 1:**
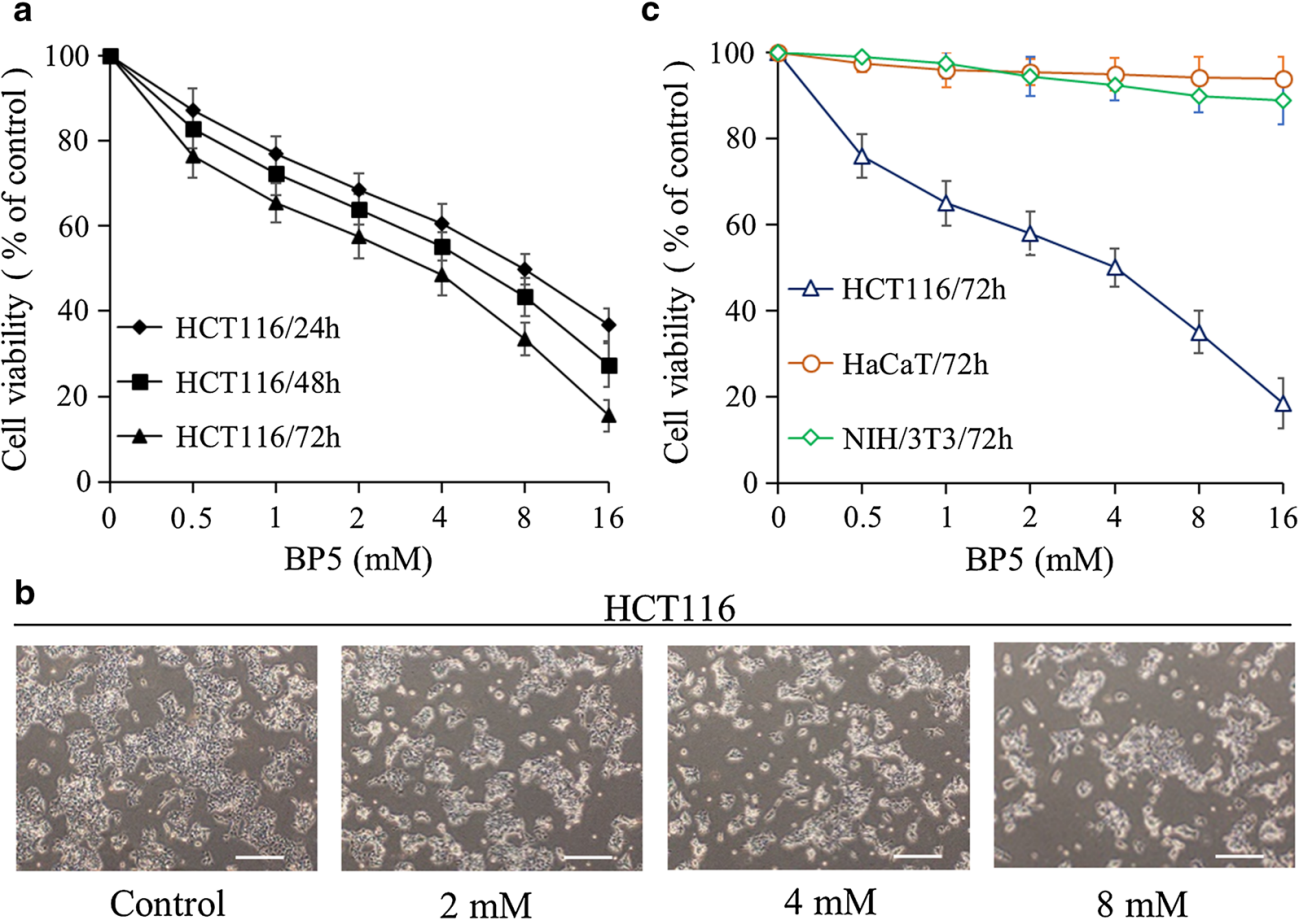
The influences of BP5 on cell viability were analysed in different cell lines. **a** BP5 inhibited cell viability in HCT116 cells. HCT116 cells were incubated with the suggested doses of BP5 for 24 h, 48 h or 72 h. Then the cell viability was examined by using a CCK-8 kit. **b** Typical images of morphological alterations of BP5-treated HCT116 cells were obtained under ×40 magnification (scale bar, 0.1 mm). **c** The effects of BP5 on the cell growth of HCT116, HaCaT and NIH/3T3 cells. Cells were cultured with the indicated doses of BP5 for 72 h. Then the CCK-8 assay was carried out to examine the cell viabilities

To investigate the anticancer activity of BP5, CCK-8 assays were performed. Cell viability was investigated after HCT116 cells were incubated with various doses of BP5 (0.5–16 mM) for 24, 48 and 72 h. As shown in Fig. 1a, the cell viability of HCT116 was significantly suppressed in a dose- and time-dependent manner. Based on the CCK-8 assay results, we selected 2, 4 and 8 mM concentrations of BP5 for further experiments. We also detected the anti-growth activity of BP5 in HCT116 cells using optical photography. The microscopic examination showed that the morphology of BP5-treated cells was deformed, exfoliated, shrinking and dead, whereas dense cells were observed in the control groups. Meanwhile, the distorted cells were increased in a concentration-dependent manner in BP5-treated cells (Fig. 1b). Cell viability analyses of HaCaT and NIH/3T3 cells were also performed after cells were cultured with varying concentrations (0.5–16 mM) of BP5 for 72 h. The data showed that BP5 had no significant inhibitory influence on cell proliferation in HaCaT and NIH/3T3 cells (Fig. 1c). The microscopic examination showed dense organization and attachment in the BP5-treated HaCaT and NIH/3T3 cells (data not shown).

### BP5 arrest cell cycle at the G1 phase

**Fig. 2:**
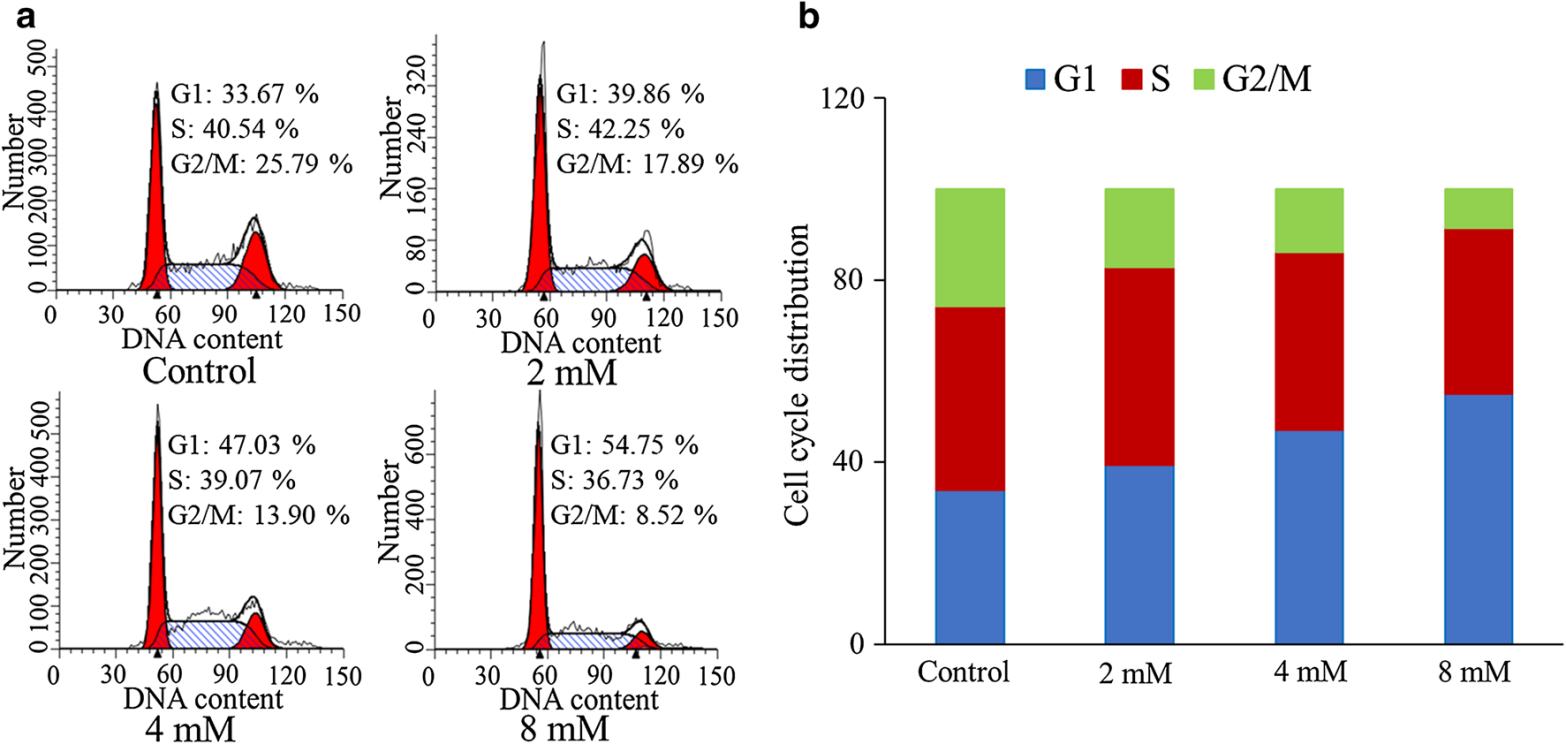
BP5 arrested cell cycle at G1 phase. **a** Representative cell cycle distribution of HCT116 cells with BP5 treatment (2, 4 and 8 mM) for 24 h. **b** The quantitative cell cycle distribution data. The quantitative data shown are mean ± SD (n = 3)

We then investigated whether BP5 has a potential to arrest cell cycle in HCT116 cells. Figure 2 shows that BP5 arrested cell cycle at G1 phase and this effect was dose-dependent in HCT116 cells. As the increase of BP5 concentration, the percentage of cells in G1 phase markedly increased, while the percentage of cells in G2/M phase decreased significantly.

### BP5 induces cell apoptosis in HCT116 cells

**Fig. 3:**
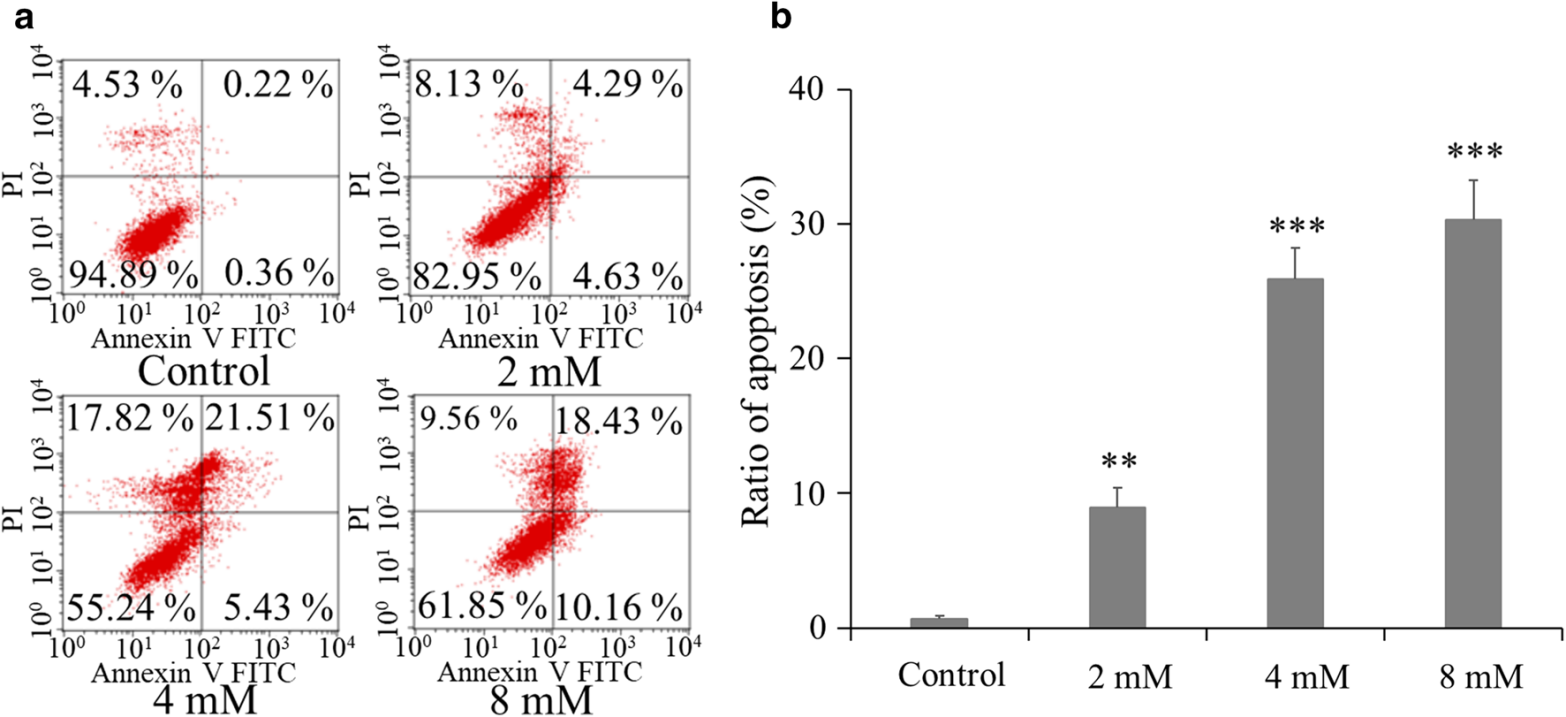
BP5 induced apoptosis in HCT116 cells. **a** Typical images of cell apoptosis distribution of HCT116 cells with BP5 treatment (2, 4 and 8 mM) for 24 h. **b** Quantitative analysis of cell apoptosis in HCT116 cells. The quantitative data shown are mean ± SD (n = 3) (**p < 0.01, ***p < 0.001)

It is well known that cell growth inhibition and apoptosis are closely linked to each other. This led us to further investigate whether BP5 could induce apoptosis in HCT116 cells. Compared with the control group, the BP5-treated groups showed significantly higher levels of apoptosis. Besides, our study also showed a significant dose-dependent increase of apoptosis after BP5 treatment in HCT116 cells (Fig. 3a, b).

### BP5 induces cell cycle arrest through its effects on cell cycle protein expression levels

**Fig. 4:**
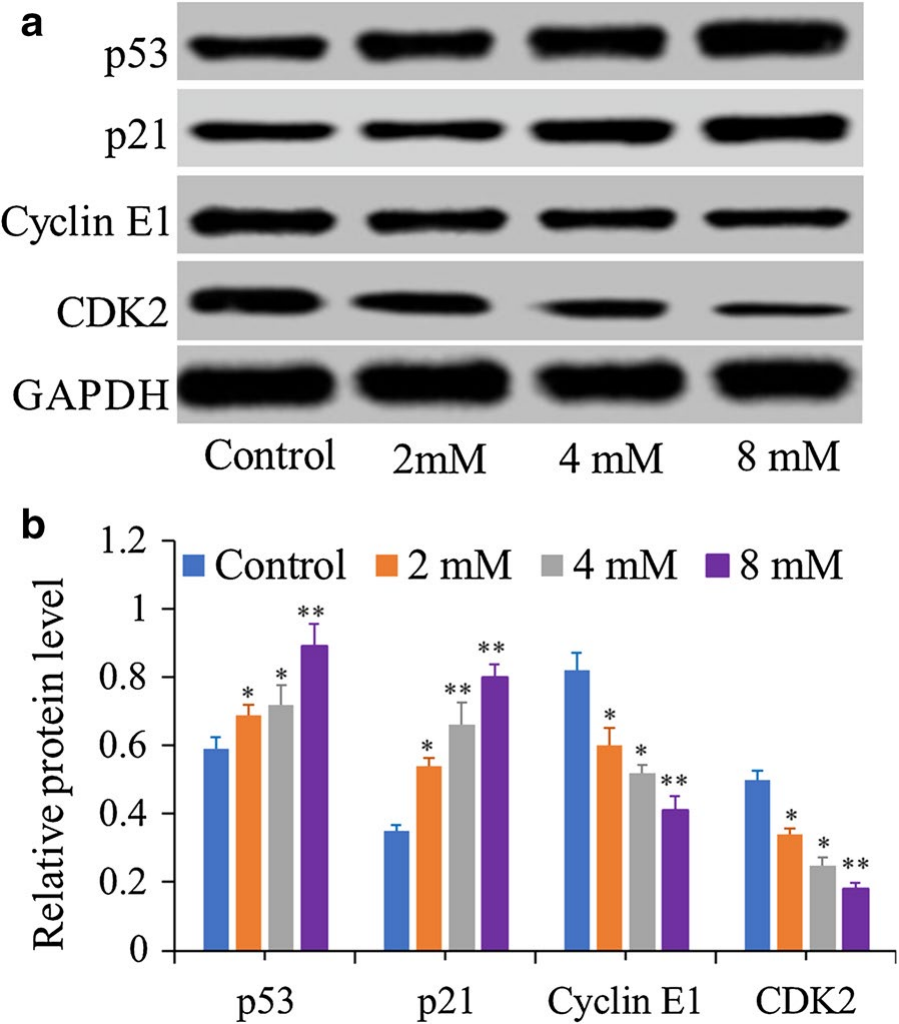
Western blot analysis of the cell cycle–related proteins in HCT116 cells. Cells were cultured with various doses of BP5 (2, 4 and 8 mM) for 24 h. **a** Representative bands of p53, p21, CyclinE1 and CDK2 from the western blot analysis. **b** Densitometry analysis of p53, p21, CyclinE1 and CDK2. The quantitative data shown are mean ± SD (n = 3) (*p < 0.5, **p < 0.01)

As showed in Fig. 4, p53 and p21 proteins were markedly elevated in the BP5-treated HCT116 cells when compared to those of the control cells. Meanwhile, in the BP5–treated HCT116 cells, the cycle arrest proteins Cyclin E1 and CDK2 were observed to decrease in expression in a dose–dependent manner.

### BP5 activates ER stress and promotes ROS generation and cytosolic Ca^2+^ production

**Fig. 5:**
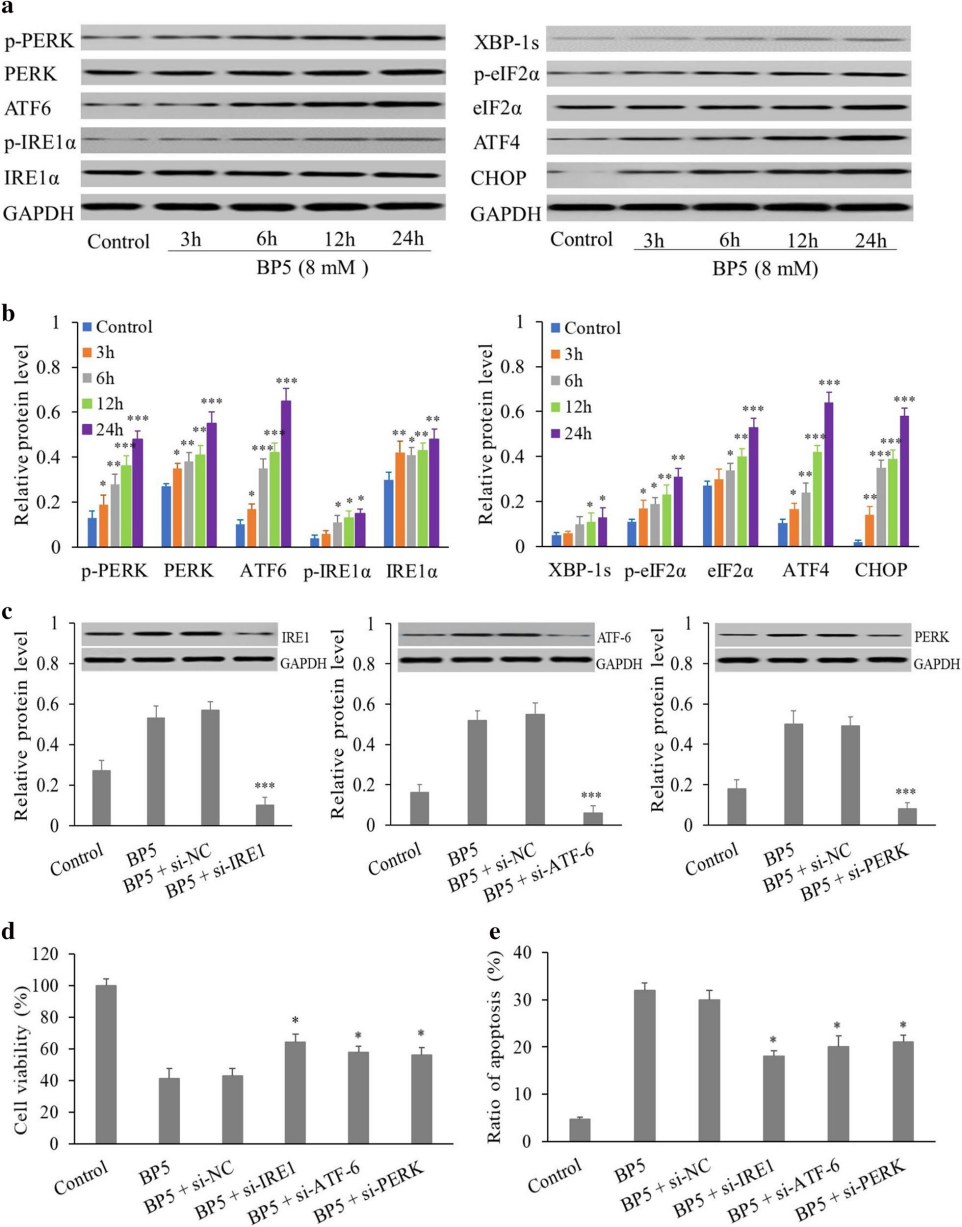
ER stress was stimulated by BP5 in HCT116 cells. **a**, **b** Western blot analysis of ER stress–associated proteins. HCT116 cells were treated with 8 mM BP5 for the indicated durations (3, 6, 12 and 24 h). Then, the cell total proteins were obtained for Western blotting. One of three representative experiments is presented. The data shown are the mean ± SD (n = 3) (*p < 0.5, **p < 0.01, ***p < 0.001 vs. control). **c**–**e** Knockdown IRE1, ATF-6, and PERK regulates oncogenic phenotypes of HCT116 cells. HCT116 cells were transfected with specific siRNAs targeting IRE1, ATF-6, and PERK, or non-targeting siRNA (si-NC), and treated with BP5 (8 mM) for 24 h. Then, western blotting analysis were used for detection the levels of IRE-1, ATF6 and PERK expressions, CCK-8 assays were performed to evaluate the cell viabilities and flow cytometry was used to detect the apoptosis (*P < 0.05, ***P < 0.001 vs. BP5-treated group)

**Fig. 6:**
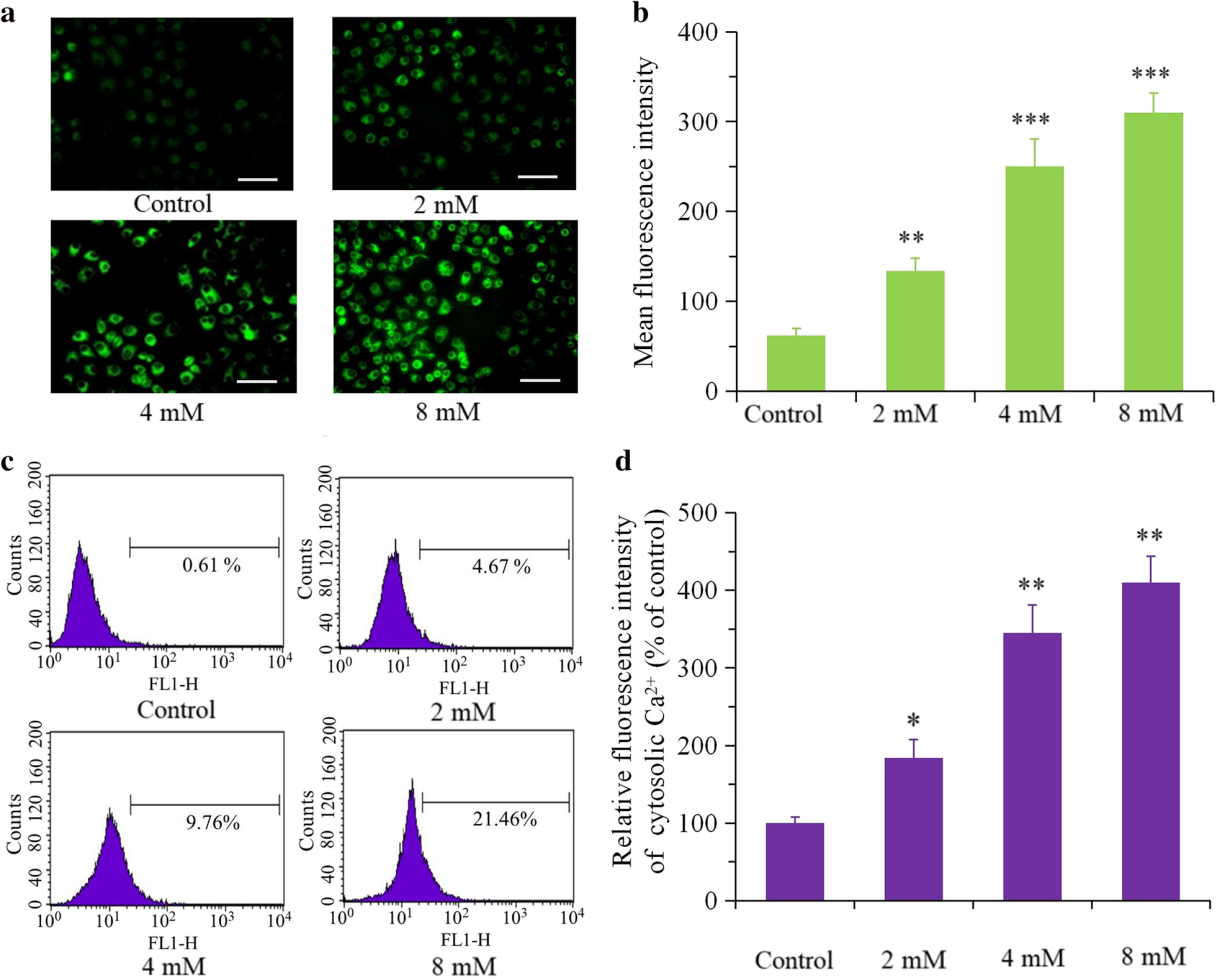
Levels of intracellular ROS and cytosolic Ca^2+^ were elevated by BP5 in HCT116 cells. HCT116 cells were cultured with various doses of BP5 (2, 4 and 8 mM) for 24 h. **a** ROS production were quantified by using the H_2_DCF-DA green fluorescence probe. Image observation were carried out by a fluorescence microscopy under ×40 magnification (scale bar, 0.1 mm). **b** Using ImageJ software, the mean fluorescence intensities of DCF were quantified (**p < 0.01, ***p < 0.001 vs. control). **c** The levels of cytosolic Ca^2+^ were quantified using Fluo-3 AM fluorescence probe. **d** The quantitative data of the levels of cytosolic Ca^2+^ shown are mean ± SD (n = 3) (*p < 0.5, **p < 0.01 vs. control)

It was reported that ER stress exerts a vital role in the cell apoptotic progresses [17–19]. High levels of ROS and elevated cytosolic Ca^2+^ can trigger ER stress-induced apoptosis [20, 21]. Therefore, we measured ER stress pathway-associated proteins and assessed ROS generation as well as cytosolic Ca^2+^ elevation in BP5-treated or -untreated HCT116 cells. As presented in Fig. 5a, b, BP5 treatment lead to the upregulation of IRE1α, ATF 6 and PERK in HCT116 cells. After 3 h of BP5 treatment, we also observed the upregulation of the phosphorylation of IRE1α and PERK. IRE1α phosphorylation resulted in XBP-1s upregulation. Correspondingly, PERK phosphorylation resulted in phosphorylation of the eIF2a subunit, p-eIF2a activated ATF4, and ATF4 further increased CHOP. These increases in expression and phosphorylation persisted until the end of study at 24 h. In particular, the increase of CHOP indicated that ER stress boosted apoptosis in the cells. Additionally, as presented in Fig. 5c, IRE1, ATF6, and PERK depletion with specific siRNAs in BP5-treated cells significantly impaired the BP5-induced expression of IRE1, ATF6, and PERK. The data also revealed that inhibition of IRE1, ATF-6 and PERK in BP5-treated groups showed higher cell viability (Fig. 5d) and lower apoptosis (Fig. 5e) compared with the BP5-only groups. In addition, BP5 treatment also caused a significant increase in ROS generation (Fig. 6a, b) and cytosolic Ca^2+^ elevation (Fig. 6c, d) in a dose-dependent manner.

### BP5 induces reduction of mitochondrial membrane potential (ΔΨ) and triggers mitochondria-mediated intrinsic apoptosis

**Fig. 7:**
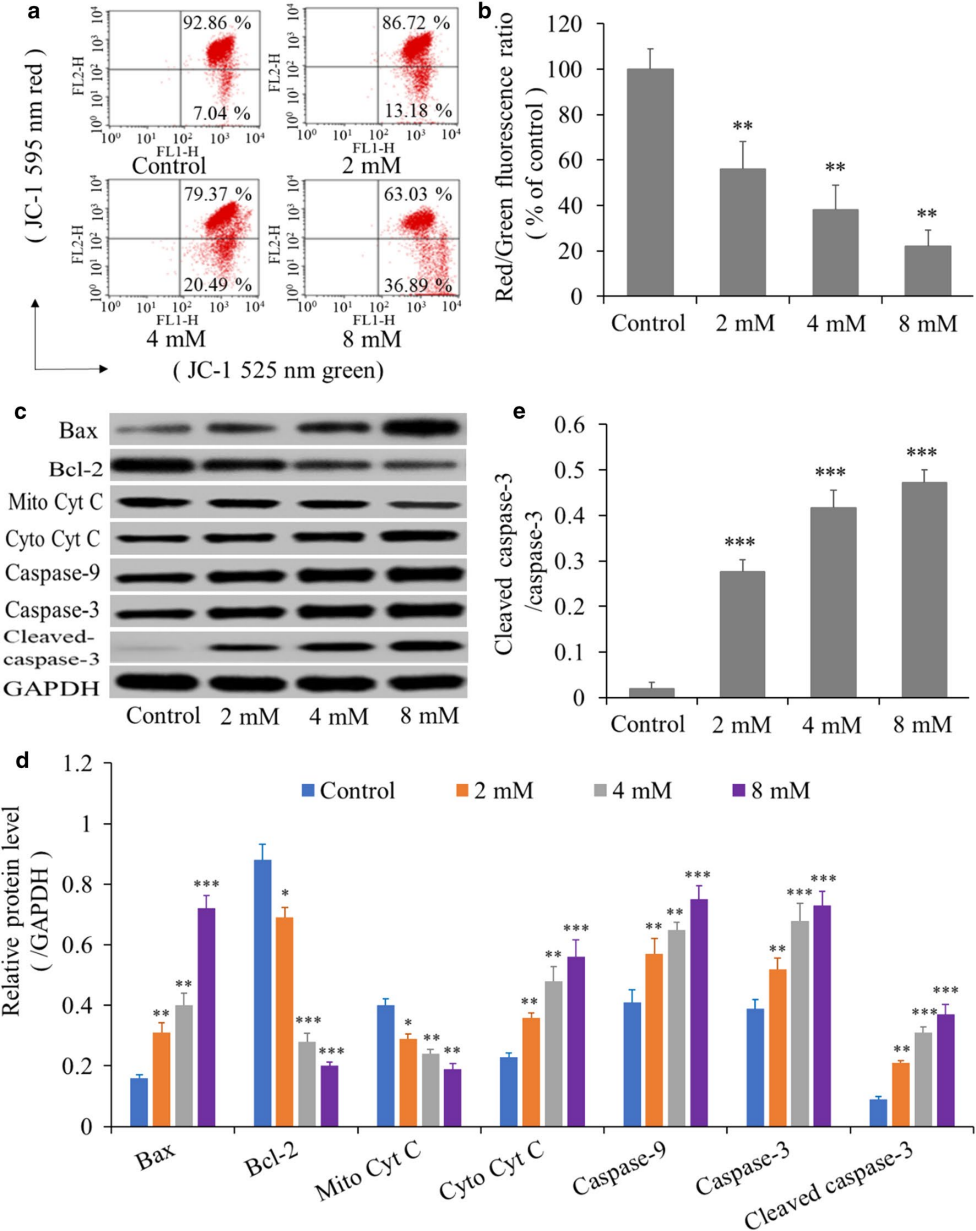
Mitochondria-mediated apoptosis was stimulated by BP5 in HCT116 cells. Cells were cultured with BP5 (0, 2, 4 and 8 mM) for 24 h. **a** Flow cytometry analysis showed the distribution of JC-1 green positive cells with lower ΔΨm (lower right quadrant). **b** Quantitative analysis of the ratio of red to green fluorescence. **c** Western blot analysis was applied to quantify the levels of Bax, Bcl-2, Mito Cyt C, Cyto Cyt C, caspase-9 and -3 as well as cleaved caspase-3. **d** Densitometry analysis of Bax, Bcl-2, Mito Cyt C, Cyto Cyt C, caspase-9 and -3 as well as cleaved caspase-3. **e** Quantitative analysis of the ratio of cleaved caspase-3 and caspase-3. The quantitative data shown are mean ± SD (n = 3) (*p < 0.5, **p < 0.01, ***p < 0.001)

Considering that mitochondria act as a central in the cell apoptotic progresses [22], we further evaluated ΔΨm and mitochondrial pathway-related proteins in BP5-treated HCT116 cells. As shown in Fig. 7a, b, BP5 treatment resulted in a significant higher green-to-red fluorescence ratio, indicating the number of cells with lower ΔΨm was increased. Bcl-2 family members serve as anti-apoptotic proteins and exhibit essential roles in controlling cell fate. On the other hand, proteins of Bax and Cyt C serve as pro-apoptotic proteins in cell apoptotic progression [23]. In this study, accompanied with increase of Bax expression, expression of Bcl-2 was decreased when HCT116 cells were treated with BP5. Meanwhile, the release of Cyt C into the cytoplasm was raised. BP5 treatment also elevated levels of caspase-9 and -3 as well as cleaved caspase-3 (Fig. 7c, d). Additionally, Western blotting results also indicated that the ratio of cleaved caspase-3/total caspase-3 was significantly raised (Fig. 7e).

### BP5 induces apoptosis in a caspase-dependent manner

**Fig. 8:**
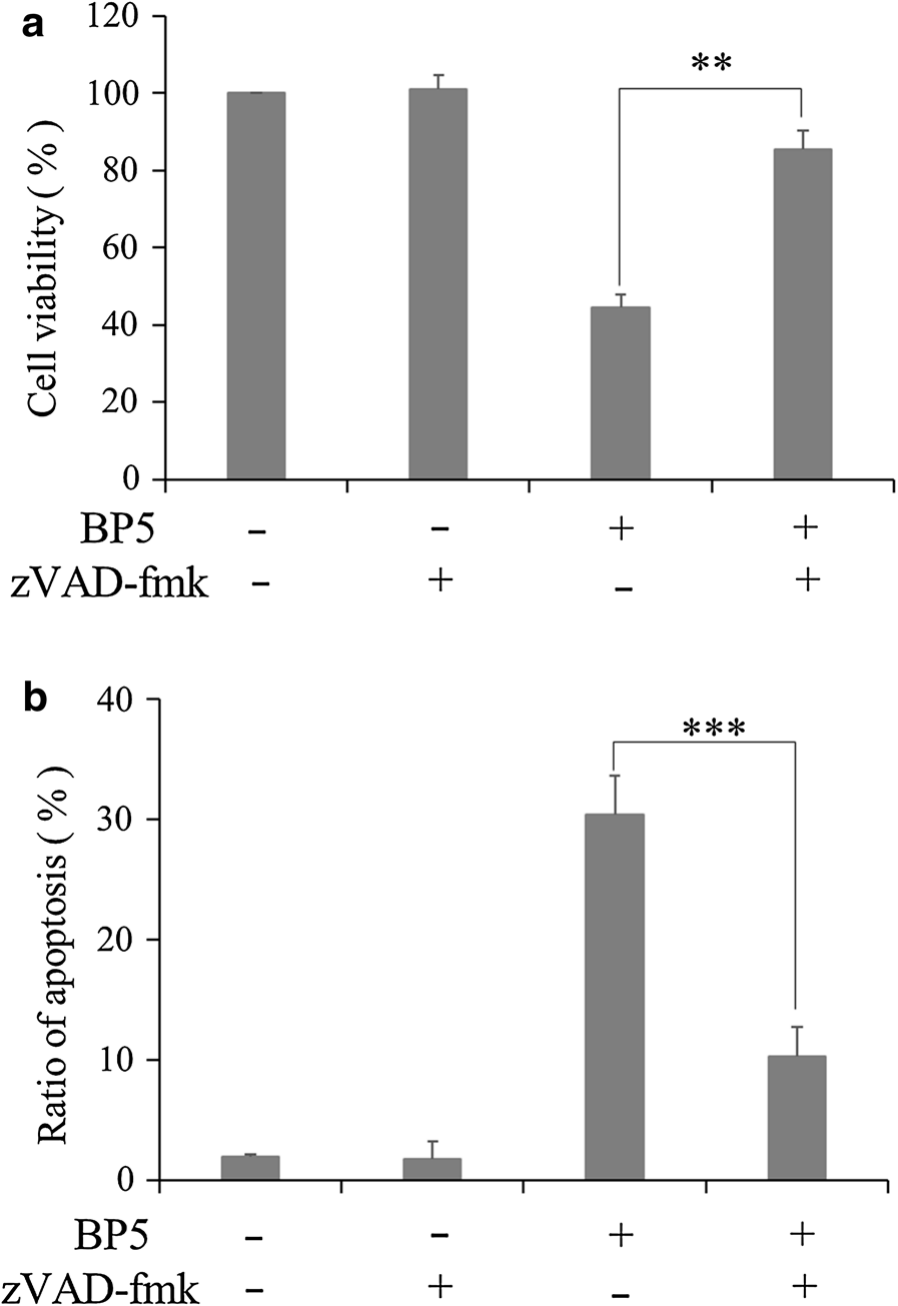
Caspase-dependent apoptosis was stimulated by BP5 in HCT116 cells. Cells were incubated with 20 μM zVAD-fmk for 2 h, then the cultures were treated with 8 mM BP5 for another 24 h. **a** Quantification of the cell viability determined by CCK8 assay. **b** Quantification of apoptotic cells detected by flow cytometry. The quantitative data shown are mean ± SD (n = 3) (**p < 0.01, ***p < 0.001)

Using zVAD-fmk (20 μM), a pan caspase inhibitor, to treat the HCT116 cells for 2 h before exposure to 8 mM BP5 for 24 h, we further examined whether BP5-induced apoptosis was caspase dependent. In this study, the influence of zVAD-fmk on cell viability in BP5- treated HCT116 cells were determined, and the effects of zVAD-fmk on BP5-induced apoptosis in HCT116 cells were also detected. As shown in Fig. 8a, b, treatment with zVAD-fmk had a little effect on the cell viability and cell apoptosis in HCT116 cells. In contrast, pretreatment with zVAD-fmk could significantly rescue the cell viability and reduce apoptosis in BP5-treated HCT116 cells.

## Discussion

In this research, we evaluated the effects of BP5, a natural pentapeptide found in the chicken bursa of Fabricius (BF), on cell proliferation and apoptosis in HCT116 cells, as well as the possible underlying signalling pathways and mechanisms. The results showed that BP5 suppressed cell viability by inducing G1 phase cell cycle arrest and apoptosis. Further investigation revealed that BP5 arrested cell cycle at G1 phase through p53 and p21 upregulation as well as Cyclin E1-CDK2 downregulation. Furthermore, BP5-mediated cell apoptosis was closely associated with the ER stress pathway via a mechanism involving activation of UPR sensors (IRE-1, PERK and ATF6) as well as their downstream signaling molecules (XBP-1s, eIF2α, ATF4 and CHOP), which was accompanied by increases in ROS generation and cytosolic Ca^2+^ production. Additionally, BP5 promoted cell apoptosis through the mitochondria-mediated apoptotic pathway via elevation of the Bax level and inhibition of the Bcl-2 level, leading reduction of ΔΨm, causing cytochrome c outflow from mitochondria and trigging caspase-9 and -3 activation. Finally, the apoptotic activation of BP5 was primarily associated with the caspase-dependent pathway.

Herein, we first assessed the anticancer ability of BP5 in HCT116 cells. The results showed that BP5 exerts anticancer potential on HCT116 cells by reducing the cell viability. It is well known that p53 exerts an important influence on the induction of cell cycle arrest [24]. As a cell proliferation inhibitor, p21 acts as an important downstream target of p53 transcriptional regulation. By inhibiting the activities of Cyclin-CDK complexes, p21 has a vital effect on cell cycle arrest at G1 phase [25]. Some reports have also revealed that G1 phase cell cycle arrest was controlled by Cyclin E-CDK2 complexes [25, 26]. In this research, our data showed that BP5 treatment dramatically elevated the levels of p53 and p21 and downregulated the levels of Cyclin E1 and CDK2 in HCT116 cells, all of which are associated with G1 phase cell cycle arrest. All data suggest that BP5 can inhibit HCT116 cell growth at least in part by modulating cell cycle progress.

Apoptosis is another main anti-cancer pathway that inhibit cancer cell proliferation in addition to cell cycle arrest. Hence, we researched whether BP5 could trigger apoptosis in HCT116 cells. Our data showed that BP5 treatment can significantly induce apoptosis in HCT116 cells. Thus, BP5 appears capable of inhibiting HCT116 cell growth also via an apoptotic mechanism. Therefore, we further researched the possible mechanisms underlying BP5-induced cell apoptosis, focusing our attention on the effects of BP5 on the ER stress-mediated apoptosis and the intrinsic mitochondrial apoptosis and the possible relationship between them.

There are lots of biochemical and physiological stimuli that can influence ER homeostasis and cause ER stress. These responses subsequently result in unfolded protein as well as misfolded proteins that are translocated into the ER [27]. High levels or persisted activation of UPR signalling is associated with cell death [28]. Therefore, the degree or duration of the UPR induced by sensors of ER stress, such as IRE-1, PERK and ATF6, seems to be more crucial for cell survival or death [29]. Under ER stress, cleaved ATF6 (cleaved from membrane-bound ATF6) is entered into cell nucleus and regulates UPR associated gene transcription. IRE1α and XBP-1s (a cleavage target of p-IRE1a) play critical roles in the ER stress [30]. The UPR also can induce phosphorylation eIF2α via PERK mediation. The p-eIF2α protein subsequently up-regulates the expression of ATF4 [31]. During ER stress-induced apoptosis, ATF-4 expression can activate pro-apoptotic signalling pathways through initiation of its downstream transcriptional factor CHOP, which subsequently triggers ER stress-specific cascades for the induction of cell apoptosis [32, 33]. Thus, to investigate the possible signalling pathways involved in BP5-induced ER stress-related apoptosis, we screened UPR sensors (IRE-1, PERK and ATF6) as well as their downstream signalling molecules (XBP-1s, eIF2α, ATF4 and CHOP) in HCT116 cells. Our data revealed that activation of these ER stress-related proteins was associated with cell growth inhibition and apoptosis elevation. Additionally, to determine the nature of IRE-1, ATF6 or PERK activity in BP5-mediated ER stress, IRE1, ATF6, and PERK signaling were blocked by using specific siRNAs. Silencing IRE1, ATF-6 and PERK decreased the expression of IRE1, ATF6, and PERK induced by BP5, as well as altered the phenotypes of BP5-treated HCT116 cells. The data indicated that BP5 activated ER stress in HCT116 cells by intensifying the natural activity of IRE-1, ATF6 and PERK signalling. It is well known that there is a closely relation between oxidative stress and ER stress [34, 35]. Previous report also revealed that higher levels of intracellular Ca^2+^ contributes to ER stress [36]. Considering that other researchers have revealed that ROS production can induce cell apoptosis via ER stress signalling [37] and that elevated-levels of cytosolic Ca^2+^ was deemed an important indicator of ER stress [38], we assessed whether ROS generation and cytosolic Ca^2+^ elevation were associated with BP5-induced ER stress. The data showed that treatment with BP5 caused a significant increase in intracellular ROS generation and cytosolic Ca^2+^ elevation. All the above data show that BP5 has an ability to induce ER stress and trigger ER stress-related pro-apoptotic signalling. Therefore, the results suggest that the ER stress signalling pathway is at least partially involved in BP5-induced HCT116 cell apoptosis.

To investigate whether the mitochondrial signalling pathway is associated with BP5-induced apoptosis, the Δψm and mitochondrial apoptosis-related proteins in HCT116 cells were measured. Our results indicated that BP5 treatment decreased the Δψm. The changes in apoptotic protein expressions is one of the pivotal events in the cell apoptosis involving mitochondrial dysfunction. The Bcl-2 family members regulates mitochondria-mediated apoptosis via delicate balance between the anti- and pro-apoptotic proteins, which plays a major role in controlling cell life and death [39]. Under stress, Bax integrates into mitochondrial membrane, and then improves the membrane permeability and induce the outflow of cyto-chrome c by forming pores on the mitochondrial membrane [40]. This mechanism permits cytochrome c to be released into cytoplasm and subsequently causes caspase activation [41]. In this study, our data showed that BP5 treatment led to Bax upregulation, Bcl-2 downregulation, cytochrome c elevation in cytosol, and caspase-9 and -3 activation in HCT116 cells. All the results indicated that the BP5-induced apoptosis of HCT116 cells occurs also through the intrinsic mitochondrial signalling pathway.

It was well known that ER-mitochondria can exchange Ca^2+^ and mediate apoptotic signal transduction [42, 43]. Some reports have revealed that Bax not only interferes with the structure and function of mitochondria, but also enhances the storage of Ca^2+^ in ER, thus increasing the Ca^2+^ load [44–46]. Under ER stress, there is persistent release of Ca^2+^ from ER, causing an absorption of cation by the mitochondrial Ca^2+^ microdomain. This subsequently increases permeability of mitochondrial membrane and participates in decoding apoptotic signals mediated by Ca^2+^ [45]. In some types of apoptosis, Bax-induced structural and functional changes in ER and increase of Ca^2+^ levels in mitochondria act as pivotal upstream signals for promoting release of cytochrome c [47]. In addition, studies have shown that ROS is not only almost simultaneous with ER stress, but is a part of UPR components, and contributing to activation of pro-apoptotic UPR signalling. Within the cell, ROS regulates both of redox-sensitive enzymes and Ca^2+^ channels. For Ca^2+^ and ROS, there is a very delicate balance exists between the favourable and adverse effects on the mitochondria [34, 48]. Furthermore, the interaction between higher levels of ROS and higher concentrations of Ca^2+^ can induce a downregulation of Δψm and subsequently result in apoptotic activation [49, 50]. Consistent with previous reports, in the present study, our data revealed that BP5 treatment can also lead to increases in Bax, Ca^2+^ ions, and ROS in HCT116 cells. In this way, it plays a role in the mechanism behind the relationship of ER stress-mediated pathway and mitochondrial-mediated pathway in BP5-induced HCT116 cancer cell apoptosis. Additionally, in our study, pretreatment with z-VAD-fmk significantly rescued the BP5-induced decrease in cell viability and reduced the BP5-induced apoptosis in HCT116 cells. The data suggest that treatment of HCT116 cells with Z-VAD-fmk can inhibit BP5-induced caspase-dependent apoptotic cascades originating from both ER and mitochondria. Collectively, we conclude that the mechanism of BP5-induced ER stress and mitochondrial-mediated apoptosis is mainly related the caspase-dependent signalling pathway.

It is noteworthy that while BP5 induced cell cycle arrest and apoptosis in HCT116 colon cancer cells, it had significantly less cytotoxicity in normal human keratinocyte HaCaT cells and mouse fibroblast NIH/3T3 cells. Moreover, our lab found that BP5 had strong inhibitory effects on the growth of the colon cancer cell lines HT29 and SW620 (Additional file 1: Figure S1). The inhibitory actions of BP5 on HT29 and SW620 were also associated with the induction of cell cycle arrest at G1 phase (Additional file 2: Figure S2) as well as with the induction of cell apoptosis (Additional file 3: Figure S3). These characteristics strengthen the applicability of BP5 as a useful candidate reagent, which is worth additional study on its anticancer activity.

## Conclusion

**Fig. 9:**
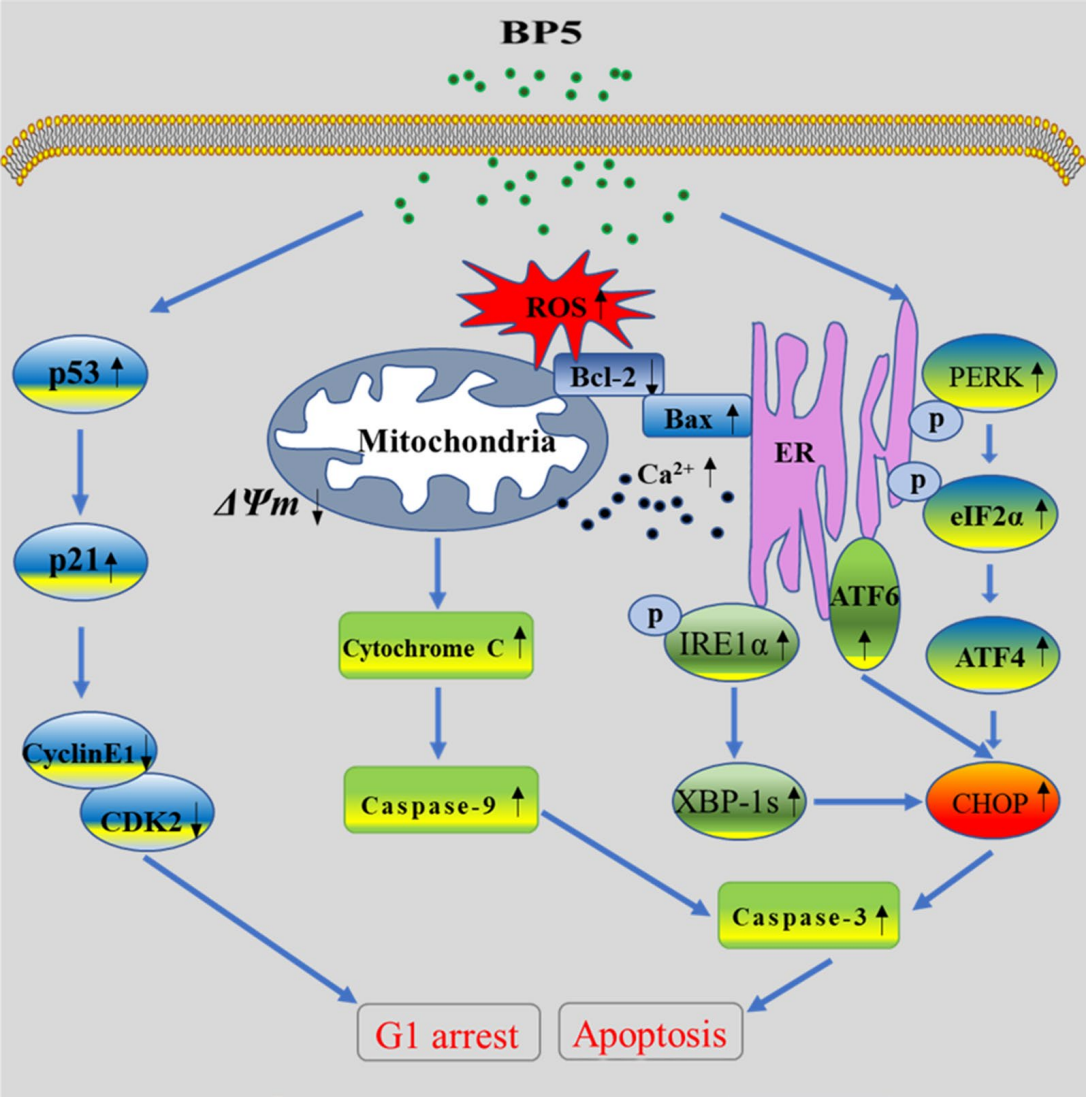
The proposed mechanisms and signalling pathways of cell cycle arrest and apoptosis induced by BP5 in HCT116 cells based on the findings of this study

The possible signalling pathways and underlying mechanisms based on the analysis of our experimental results are summarized in Fig. 9, indicating that BP5 treatment can arrest cell cycle at G1 phase and induce ER stress/mitochondria-mediated caspase-dependent apoptosis in HCT116 cells. Since this is the first study to report that BP5 has an inhibitory effect on the growth of HCT116 cells, further mechanistic studies on the anticancer activities of BP5 are required in vitro and in vivo. Our present discoveries provide insight into the further investigation on the anticancer activities of BP5.

## Additional files

**Additional file 1: Figure S1.** BP5 induced cell growth inhibition in HT29 and SW620 cells.

**Additional file 2: Figure S2.** BP5 arrested cell cycle at G1 phase in HT29 and SW620 cells.

**Additional file 3: Figure S3.** BP5 induced apoptosis in HT29 as well as SW620 cells.

## Data Availability

The data generated or analysed during this study are available within the article.

## Abbreviations

ER: endoplasmic reticulum
BP5: Bursopentin
UPR: unfolded protein response
ROS: reactive oxygen species
Cyt C: cytochrome c
ΔΨm: mitochondrial membrane potential
