# Supplementary material for: Bursopentin (BP5) induces G1 phase cell cycle arrest and endoplasmic reticulum stress/mitochondria-mediated caspase-dependent apoptosis in human colon cancer HCT116 cells
Source: Cancer Cell Int. 2019 May 16;19:130. doi: 10.1186/s12935-019-0849-3 (PMC6521404; doi:10.1186/s12935-019-0849-3)
Supplement: Supplementary file 1 — Additional file 1: Figure S1. BP5 induced cell growth inhibition in HT29 and SW620 cells. [file 12935_2019_849_MOESM1_ESM.docx]

**Additional file**

**Bursopentin (BP5) induces G1 phase cell cycle arrest and endoplasmic reticulum stress/mitochondria-****mediated caspase-dependent apoptosis** **in human colon cancer HCT116 cells**

Jing Li^1, 2^, Tian-xiang Li^3^, Yao Ma^1, 2^, Yong Zhang^1, 2^, De-yuan Li^4^ *, Hai-rong Xu ^1, 2, 5^ *

**Additional file 1**

**
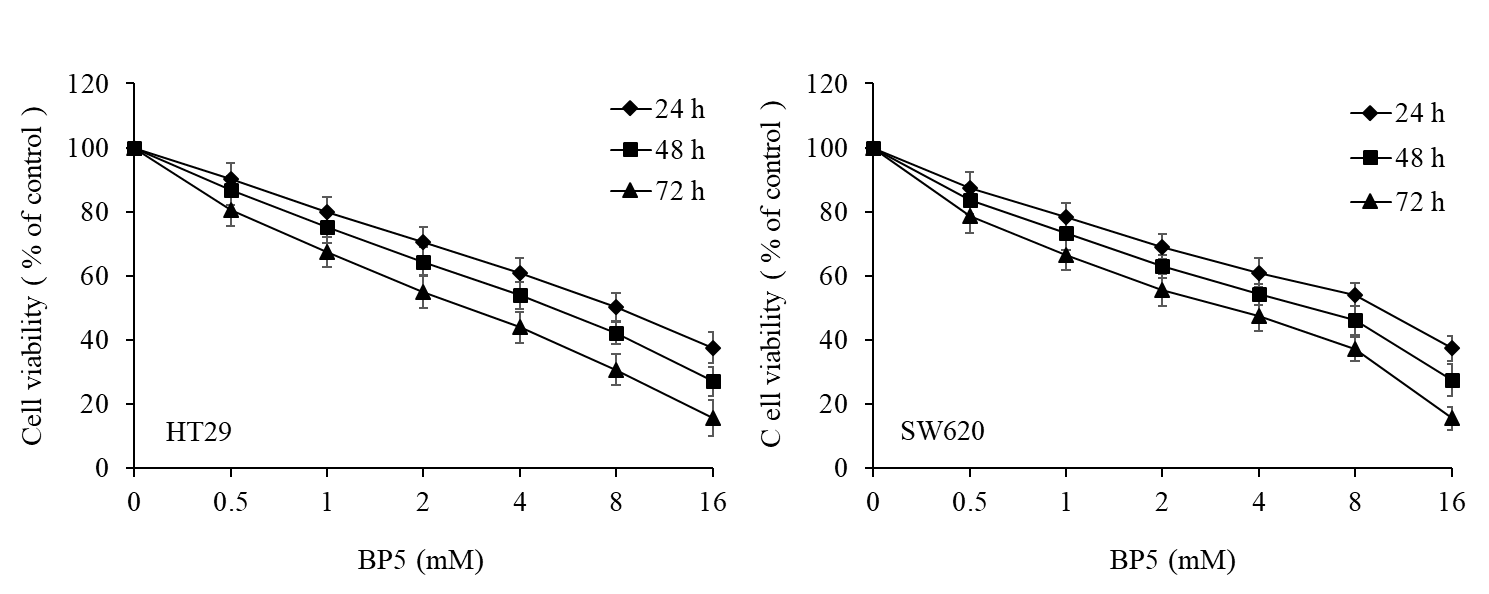
**

**Figure S1.** BP5 inhibited the growth of the human colon cancer cell lines HT29 and SW620. Cells of HT29 and SW620 were incubat­ed with the indicated concentrations of BP5 for 24 h, 48 h and 72 h, then a CCK-8 assay was performed to detect the cell vi­ability. Data represent the mean ± SD of three independent experiments.
