# Supplementary material for: Bursopentin (BP5) induces G1 phase cell cycle arrest and endoplasmic reticulum stress/mitochondria-mediated caspase-dependent apoptosis in human colon cancer HCT116 cells
Source: Cancer Cell Int. 2019 May 16;19:130. doi: 10.1186/s12935-019-0849-3 (PMC6521404; doi:10.1186/s12935-019-0849-3)
Supplement: Supplementary file 2 — Additional file 2: Figure S2. BP5 arrested cell cycle at G1 phase in HT29 and SW620 cells. [file 12935_2019_849_MOESM2_ESM.docx]

**Additional file 2**

**
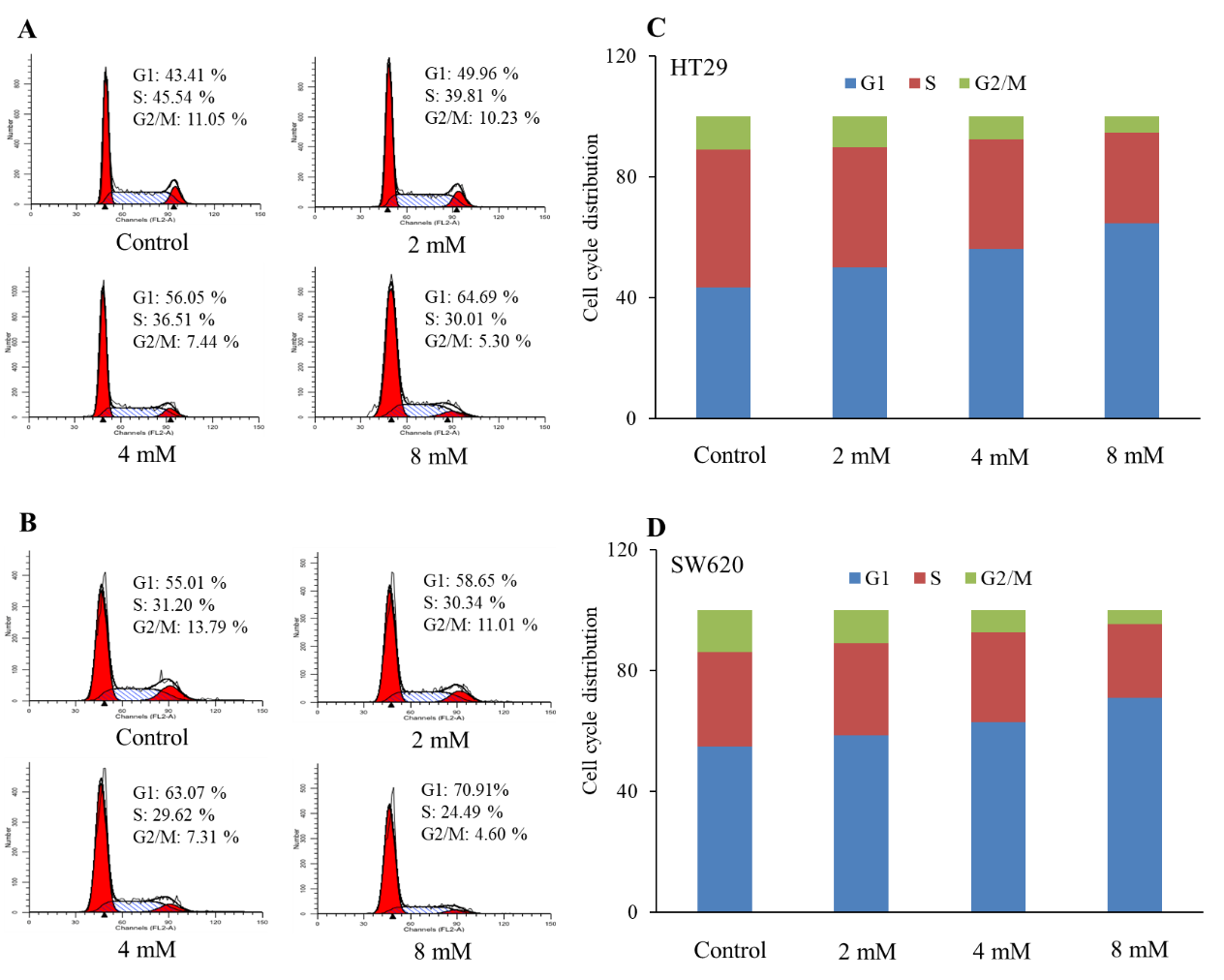
**

**Figure S2.** BP5 induced cell cycle arrest at the G1 phase in HT29 and SW620 cells. HT29 cells and SW620 cells were treated with BP5 (2, 4 and 8 mM) for 24 h, and then the cells were assessed by flow cytometry using propidium iodide (PI) staining. (**A**) and (**B**) Representative scans of the fluorescence pattern with PI staining and the cell cycle distribution of HT29 and SW620 cells. (**C**) and (**D**) Quantitative cell cycle distribution data of HT29 and SW620 cells. The quantitative data shown are the mean ± SD of three independent experiments.
