# Supplementary material for: Bursopentin (BP5) induces G1 phase cell cycle arrest and endoplasmic reticulum stress/mitochondria-mediated caspase-dependent apoptosis in human colon cancer HCT116 cells
Source: Cancer Cell Int. 2019 May 16;19:130. doi: 10.1186/s12935-019-0849-3 (PMC6521404; doi:10.1186/s12935-019-0849-3)
Supplement: Supplementary file 3 — Additional file 3: Figure S3. BP5 induced apoptosis in HT29 as well as SW620 cells. [file 12935_2019_849_MOESM3_ESM.docx]

**Additional file 3**

**
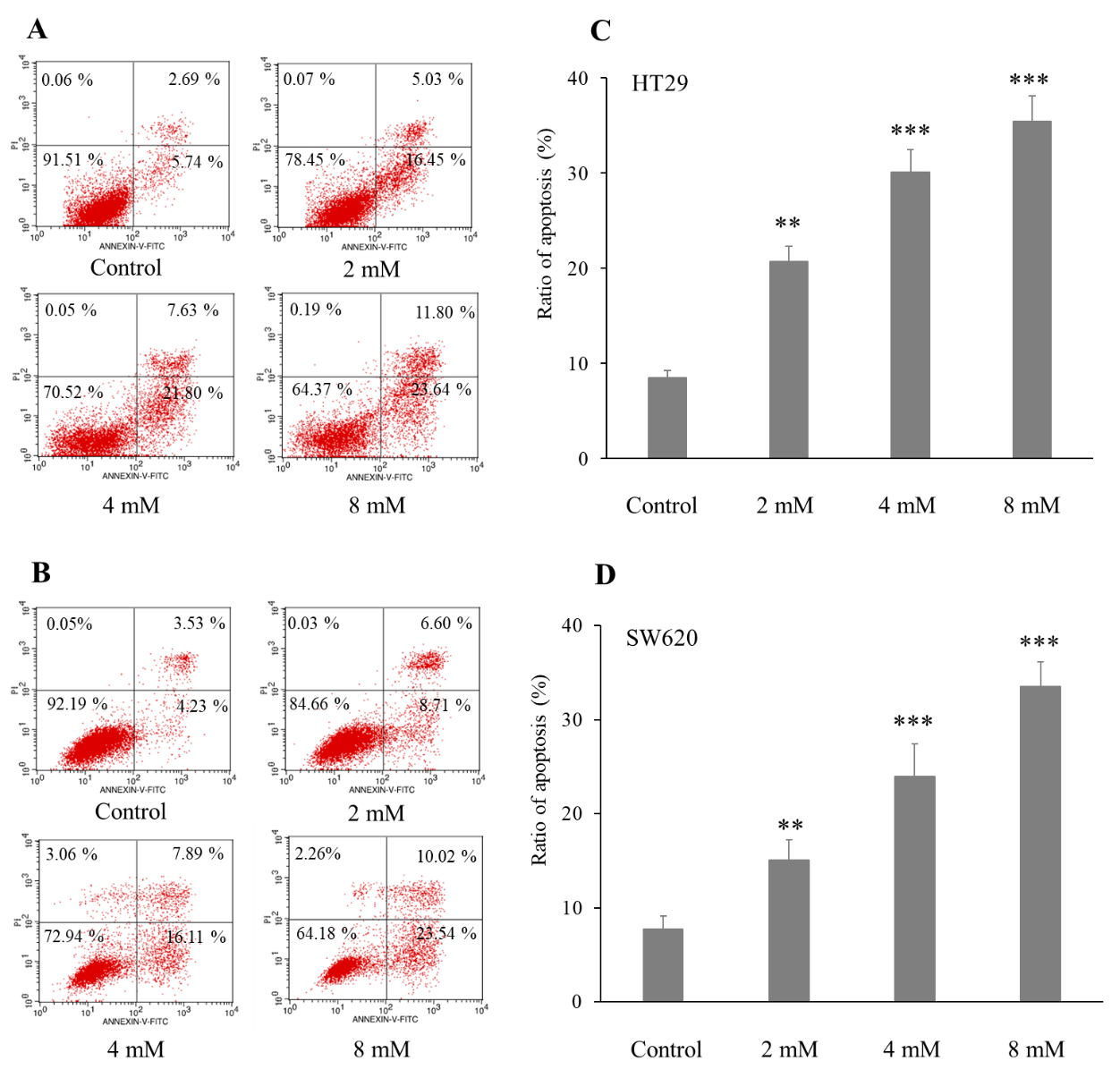
**

**Figure S3.** BP5 induced apoptosis in HT29 and SW620 cells. HT29 cells and SW620 cells were treated with BP5 (2, 4 and 8 mM) for 24 h, then the cells were assessed by flow cytometry using annexin V-FITC/PI double staining. (**A**) and (**B**) Typical images of cell apoptosis distribution in HT29 and SW620 cells. (**C**) and (**D**) Quantitative analysis of apoptosis in HT29 and SW620 cells. Data are shown as the mean ± SD of three separate experiments (***P* < 0.01, ****P* < 0.001).
